# Sexual Orientation, Femininity, and Attitudes Toward Menstruation Among Women: Implications for Menopause

**DOI:** 10.1093/geroni/igaa057.3173

**Published:** 2020-12-16

**Authors:** Jes Matsick, Mary Kruk, Britney Wardecker

**Affiliations:** The Pennsylvania State University, University Park, Pennsylvania, United States

## Abstract

Women differ in how they psychologically respond to the end of menstruation and onset of menopause (Lorber & Moore, 2002); however, little empirical evidence exists for understanding how sexual orientation and gendered dynamics contribute to menopausal experiences. Do women’s attitudes toward the cessation of menstruation vary by sexual orientation? Using data from the Midlife in the U.S. Study (MIDUS, Wave 2; N=2,951), we test how sexual orientation relates to attitudes toward menstruation cessation through norms and values surrounding womanhood (i.e., “traditional femininity concerns,” such as worries about fertility and attractiveness). Cisgender heterosexual women, compared to sexual minority women, expressed greater regret of menstrual periods ending, and heterosexual women’s heightened concerns about traditional femininity mediated the association between sexual orientation and regret, b=-.09, 95%CI [-.176,-.008]. This research yields implications for understanding aging stigma and women’s health, and we discuss how menopause may be differently experienced by women based on sexual orientation.

